# Primary biliary cirrhosis and psoriasis: a two-sample Mendelian randomization study

**DOI:** 10.3389/fimmu.2023.1264554

**Published:** 2024-01-04

**Authors:** Diqian Zhao, Qinyu Zhao, Fangwei Xu, Fang Zhang, Wenzhe Bai

**Affiliations:** ^1^ The First Clinical Medical School, Shandong University of Traditional Chinese Medicine, Jinan, China; ^2^ Institute of Acupuncture and Moxibustion, Shandong University of Traditional Chinese Medicine, Jinan, China; ^3^ Department of Radiology, The Affiliated Hospital of Shandong University of Traditional Chinese Medicine, Jinan, China; ^4^ Department of Dermatology, The Affiliated Hospital of Shandong University of Traditional Chinese Medicine, Jinan, China; ^5^ Postdoctoral Mobile Station, Shandong University of Traditional Chinese Medicine, Jinan, China

**Keywords:** primary biliary cirrhosis, psoriasis, Mendelian randomization study, genome-wide association studies, causal relationship

## Abstract

**Background:**

Primary biliary cirrhosis (PBC) and psoriasis are frequently observed to co-occur in clinical settings. However, the causal associations and underlying mechanisms between PBC and psoriasis remain poorly defined.

**Methods:**

In this study, we conducted bidirectional MR analysis to explore the causal relationship between PBC and psoriasis using four MR methods: inverse-variance weighted, MR-Egger regression, weighted median, and weighted mode. Sensitivity analyses were carried out, employing different models and testing methods for comparison to assess the influence of heterogeneity and pleiotropy on our findings and to confirm the robustness of these results.

**Results:**

A causal relationship between the risk of PBC and psoriasis was identified, as confirmed by IVW analysis (OR: 1.081, 95%CI: 1.028~1.137, *P*<0.05). The other three MR methods also produced similar results. However, psoriasis did not have a causal effect on PBC risk (OR: 1.022, 95%CI: 0.935~1.118, *P*>0.05). The intercept of MR-Egger regression was 0.0013 (*P*>0.05), indicating that genetic pleiotropy did not influence the results. Additionally, the leave-one-out analysis demonstrated the robustness of our MR findings.

**Conclusion:**

This study reveals a causal relationship between PBC and psoriasis, with PBC increasing the risk of psoriasis, but not the reverse. This potential causal relationship offers a new perspective on the etiology of PBC.

## 1 Introduction

Psoriasis is a chronic inflammatory and proliferative skin disorder. Its clinical manifestations differ according to the type, with common forms including plaque, guttate, erythrodermic, and pustular psoriasis. These types share characteristics of skin erythema, thickening, and scaling (1). A systematic analysis and modeling study using the Global Health Database indicate considerable variation in the prevalence of psoriasis among adults across different countries and regions. In Europe, the prevalence is 0.91% (95%CI: 0.29%~3.03%) (2). Psoriasis patients often experience comorbidities such as arthritis, cardiovascular disease, mental disorders, and enteritis (1). The refractory and recurrent nature of psoriasis, combined with the pruritus and pain associated with its lesions, significantly affects the physical and mental well-being and quality of life of patients.

Primary biliary cirrhosis (PBC), also known as primary biliary cholangitis, is an autoimmune condition causing chronic inflammatory damage to the liver. The exact etiology and pathophysiology of PBC remain unclear. It is characterized by chronic non-suppurative destructive cholangitis, primarily affecting the interlobular and septal bile ducts, leading to periductal inflammatory infiltration and necrosis (3). PBC predominantly affects women and individuals over 50 years of age (4). Its prevalence has tended to increase over time, likely due to advancements in diagnostic methods and better access to healthcare resources (5). A systematic review and meta-analysis of primary biliary cholangitis epidemiology in European countries showed that the global prevalence and incidence of PBC vary geographically, with a pooled prevalence of 22.27 per 100,000 population (95%CI: 17.98~27.01) (6). In individuals diagnosed with PBC, there is a notable prevalence of concurrent autoimmune disorders. These include, but are not limited to, Sjögren’s Syndrome (3.5–73%), autoimmune thyroid diseases (5.6–23.6%), systemic sclerosis (1.4-12.3%), and systemic lupus erythematosus (0–3.7%) (3). Symptoms such as cholestatic pruritus, abdominal discomfort, and fatigue significantly impair the quality of life of affected individuals (7).

PBC and psoriasis, both immune-related diseases, have an unclear relationship. Psoriasis, with its skin manifestations, adversely affects patients’ quality of life and mental health. Therefore, it is crucial for physicians to monitor the skin conditions of patients with PBC, ensuring timely detection and diagnosis of psoriasis, and to optimize treatment regimens. An international multicenter study involving 1,554 PBC patients reported that 23 cases (1.5%) had concurrent psoriasis (8). However, PBC often presents with nonspecific early symptoms, and patients may overlook mild or localized psoriatic lesions, leading to delayed diagnosis and treatment. Certain studies have indicated that both IL-23 and TL1A play roles in the pathogenesis of psoriasis and PBC, suggesting a potential association between these conditions (9, 10). Yet, conclusive evidence establishing a causal link between PBC and psoriasis is lacking, necessitating further investigation.

Traditional observational studies are limited by issues such as confounding, reverse causality, and selection bias. In contrast, Mendelian randomization (MR) is a method in genetic epidemiology that uses genetic variants as instrumental variables (IVs) for causal inference between exposures and outcomes (11). These genetic variants, randomly assorted during meiosis and fixed at conception, are long-term stable exposure factors unaffected by environmental or social factors, thereby overcoming the limitations of observational studies. In this research, we employed a bidirectional MR analysis to investigate whether PBC and psoriasis influence each other genetically.

## 2 Materials and methods

### 2.1 Data source

To investigate the causal relationship between PBC and psoriasis, we utilized single nucleotide polymorphisms (SNPs) as instrumental variables, sourced from genome-wide association studies (GWAS) databases. Given the public nature of these databases, additional ethical approval was not required. The PBC-related database encompasses genotype data from 2,861 PBC cases and 8,514 controls, all from European populations, along with association analysis results for 119,756 SNPs (GWAS ID: ebi-a-GCST005581) (12). This database originates from a GWAS published in Nature Genetics, which focused on uncovering genetic susceptibilities and molecular mechanisms of PBC. The database related to psoriasis includes genotype data from 4,510 psoriasis cases and 212,242 controls from Finnish populations, with association analysis results for 16,380,464 SNPs (GWAS ID: finn-b-L12_PSORIASIS).

### 2.2 SNP selection

To ensure the accuracy and reliability of our research findings, we implemented stringent criteria for SNP selection. SNPs with genome-wide significant associations with the exposure (*P* < 5 × 10^-8^) were chosen, and those exhibiting high linkage disequilibrium (r^2^ > 0.001 and kb < 10,000) were excluded. In cases where the result dataset lacked exposure-related SNPs, we opted for alternative SNPs showing high correlation with the associated SNPs (r^2^ > 0.8). To maintain the accuracy of the MR analysis, it was necessary to filter out palindromic SNPs, which are SNPs with effect alleles and other alleles as complements. These rigorously screened SNPs were then employed as the final instrumental variables for the Mendelian randomization analysis that followed.

### 2.3 MR assumption

**Figure 1:**
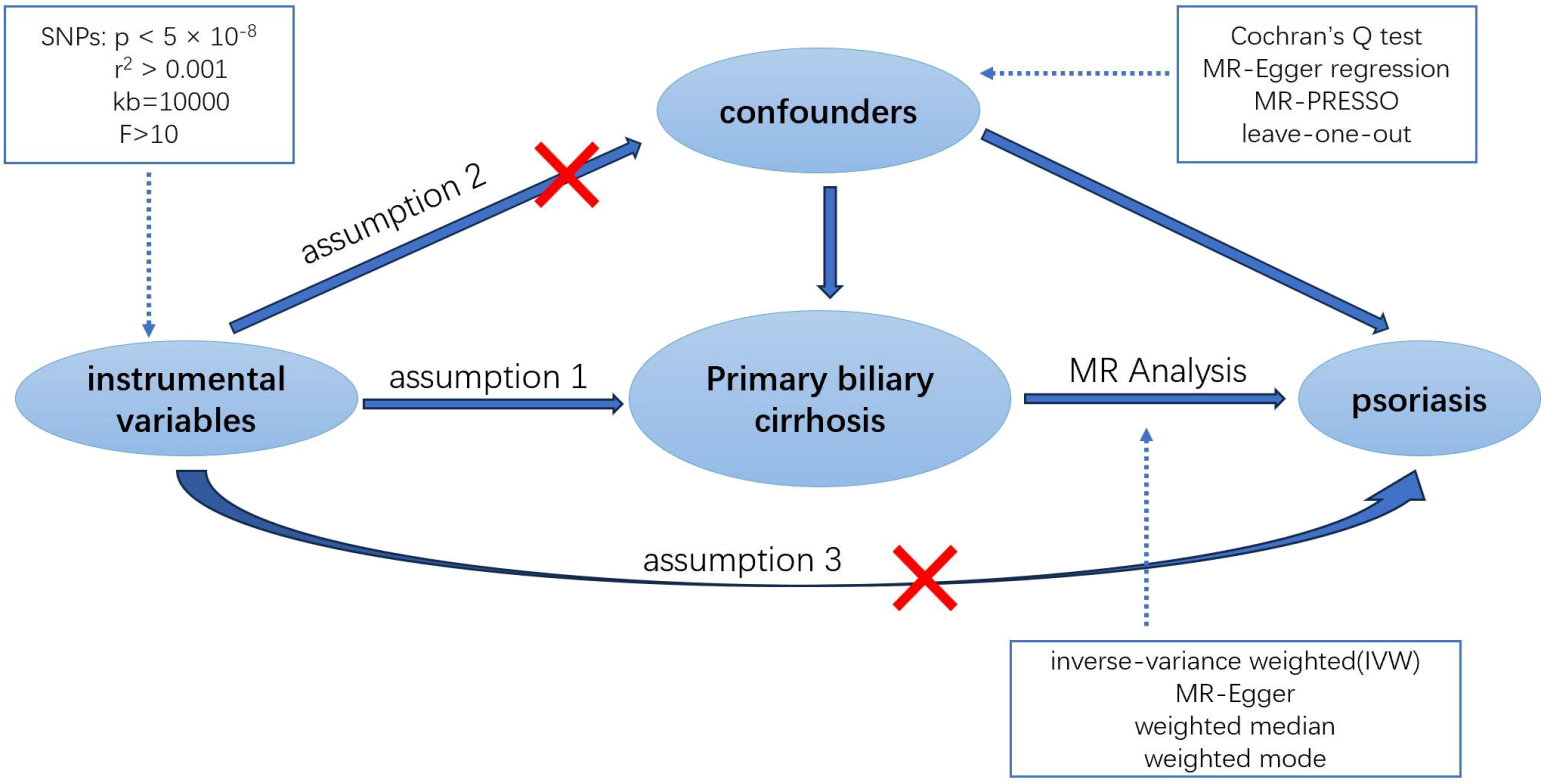
An overview of this Mendelian randomization (MR) study design.

MR analysis hinges on three core assumptions to minimize bias in the results (13): 1. Relevance Assumption: The selected IVs must be directly associated with the exposure. 2. Independence Assumption: The IVs must be independent of any confounders in the exposure-outcome association. 3. Exclusion Restriction Assumption: The IVs must influence the outcome solely through the exposure (**Figure 1**). To evaluate the strength of the association between the instrumental variables and the exposure, and to eliminate weak instrumental variables, we utilized the F-statistic (14). This statistic is calculated as F = Beta^2^/SE^2^, where Beta and SE represent the estimated effect and standard error of the allele on the exposure, respectively. Instrumental variables with an F-statistic less than 10 were excluded to avoid potential genetic confounding or measurement error.

### 2.4 Statistical analysis

In this research, we conducted a two-sample MR analysis to assess the causal relationship between PBC and psoriasis. The inverse-variance weighted (IVW) method was our primary approach, complemented by three additional methods: MR-Egger regression, weighted median, and weighted mode approaches. These methods collectively provide a comprehensive assessment of the potential relationship. The IVW method, characterized by excluding the intercept term in regression and using the inverse of the outcome variance as the weight for fitting, offers the most accurate reference for causal inference (15). MR-Egger regression, another MR method, can detect and adjust for horizontal pleiotropy by incorporating an intercept term into the IVW method. It is used to evaluate whether the instrumental variables satisfy the exclusion restriction assumption (16). Although its precision is relatively lower, it is useful in understanding the direction and magnitude of the effect. The weighted median approach, with a lower type I error rate and higher causal estimation capability, serves as a sensitivity analysis to verify the reliability of IVW results (17). Lastly, the weighted mode approach leverages the similarity information between SNPs to enhance the precision and robustness of the estimates (18).

### 2.5 Pleiotropy, heterogeneity, and sensitivity evaluation

We employed Cochran’s Q test to examine the presence of heterogeneity among individual genetic variant estimates. In cases of significant heterogeneity (*P*<0.05), we utilized the IVW random effects model, rather than the fixed effects model, to derive the final MR results (19). To assess the impact of horizontal pleiotropy on MR analysis, we calculated the intercept value using MR-Egger intercept analysis. The MR-PRESSO test was also implemented to identify and exclude outliers influenced by horizontal genetic pleiotropy, thereby enhancing the credibility of the Mendelian randomization. Additionally, a leave-one-out analysis was performed to evaluate the influence or bias of each SNP on the combined estimate. All MR analyses and tests were conducted using the “TwoSampleMR” and “MRPRESSO” packages in R software (version 4.3.1).

## 3 Result

### 3.1 Causal effect of PBC on psoriasis from MR analysis

We extracted 22 SNPs closely associated with PBC risk, ensuring no linkage disequilibrium (r^2^ < 0.001) among them, and confirming they were not weak instrumental variables as their F-statistics all exceeded 10, aligning with our earlier selection criteria. Utilizing MR-PRESSO, we identified and removed three anomalous SNPs (rs1646019, rs3135024, and rs34725611). The remaining 19 SNPs were then used as IVs for Mendelian analysis, with their details provided in **Supplementary Table S1**. Cochran’s Q test indicated heterogeneity among the IVs, evidenced by a Q-statistic of 38.15 (*P*<0.05), leading us to employ the IVW random effects model for further MR analysis.

**Figure 2:**
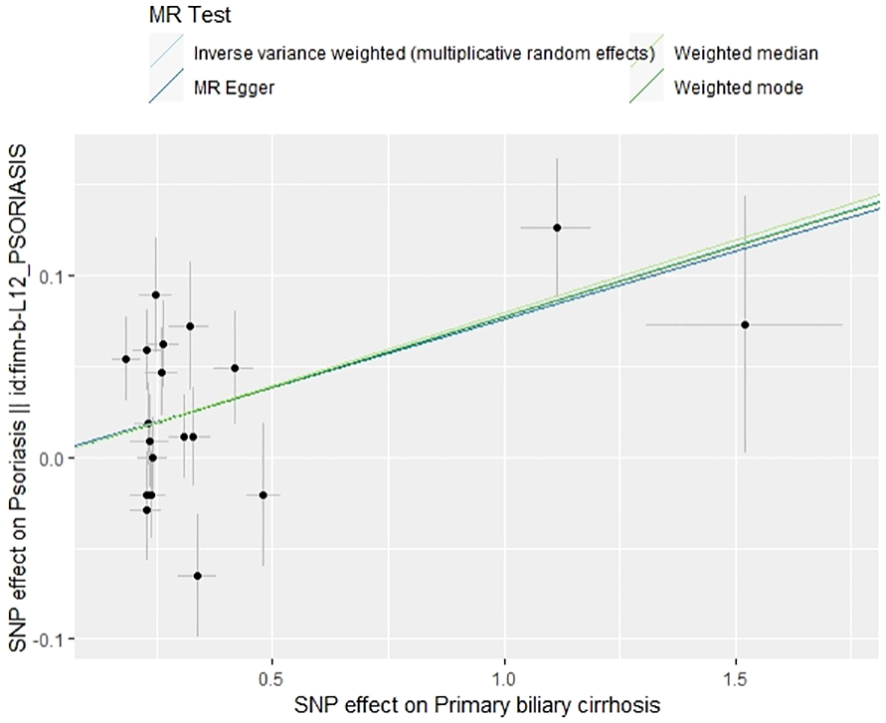
Scatter plot of the effect of PBC on psoriasis.

**Figure 3:**
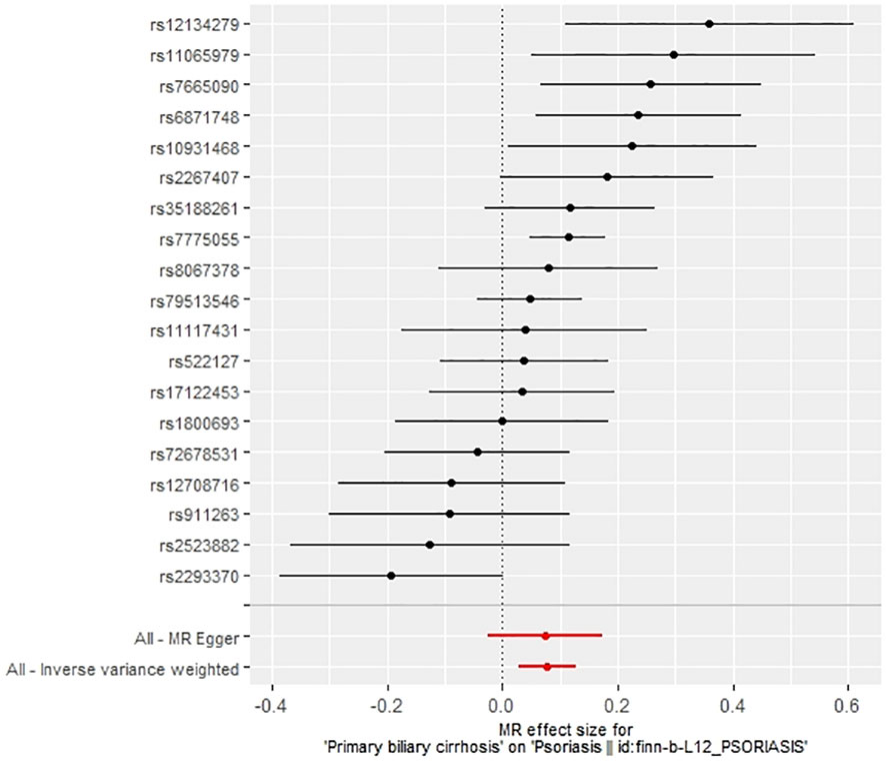
Forest plot of the effect of PBC on psoriasis.

**Figure 4:**
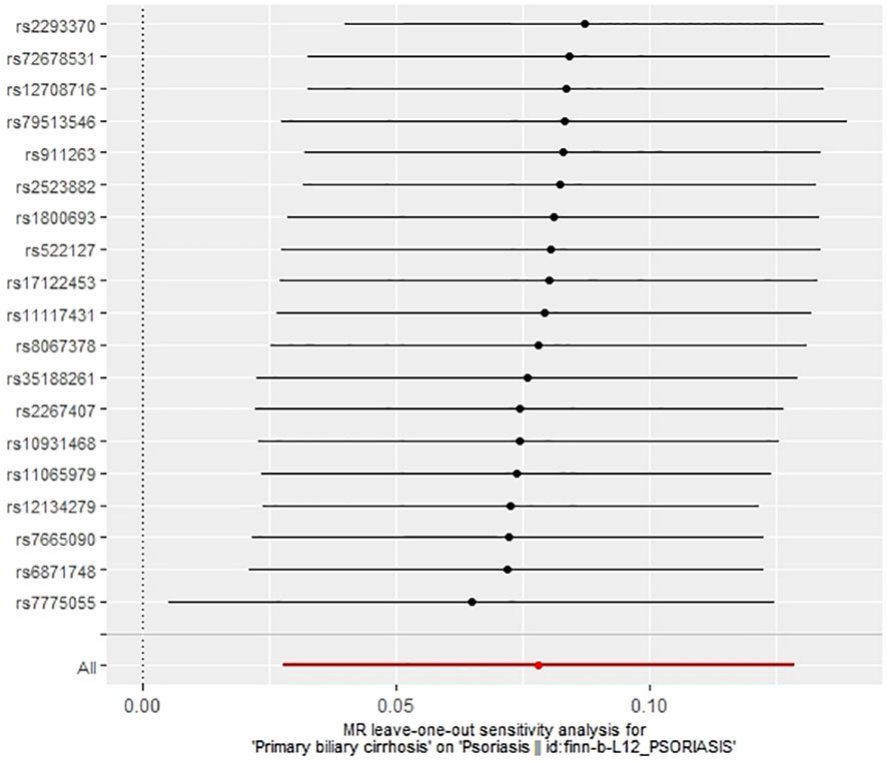
Leave-one-out plot of the effect of PBC on psoriasis.

**Figure 5:**
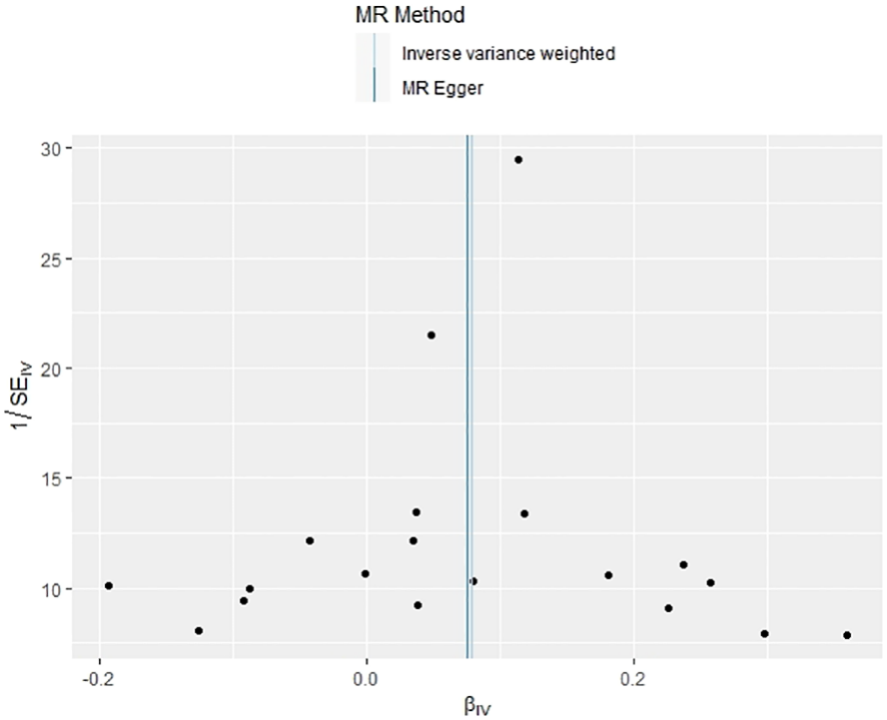
Funnel plot of the effect of PBC on psoriasis.

The IVW method revealed a causal relationship between PBC risk and psoriasis (OR: 1.081, 95%CI: 1.028~1.137, *P*<0.05). This finding was corroborated by the weighted model (OR: 1.081, 95%CI: 1.023~1.142, *P*<0.05) and the weighted median (OR: 1.083, 95%CI: 1.031~1.139, *P*<0.05), all indicating a positive causal effect of PBC on the risk of psoriasis. However, the MR-Egger analysis did not show a significant association (OR: 1.078, 95%CI: 0.977~1.190, *P*>0.05) (**Table 1**). Further examination of horizontal pleiotropy through MR-Egger intercept analysis (b-intercept: 0.0013; se: 0.0176; *P*>0.05) suggested a low probability of genetic pleiotropy effects, thus minimizing the likelihood of confounding bias impacting the analysis results. Sensitivity analysis using the leave-one-out method, which entailed sequential removal and recalculation of causal effects with the remaining SNPs, demonstrated consistent results, affirming the reliability of our findings (**Figures 2**–**5**).

**Table 1:** Mendelian randomization estimates for PBC on psoriasis.

Exposure	Outcome	No. of IVs	Methods	Beta	SE	OR (95%CI)	P
PBC	Psoriasis	19	MR Egger	0.075	0.050	1.078 (0.977~1.190)	0.154
			IVW	0.078	0.026	1.081 (1.028~1.137)	0.002
			Weighted mode	0.078	0.028	1.081 (1.023~1.142)	0.012
			Weighted median	0.080	0.025	1.083 (1.031~1.139)	0.002

IVs, instrumental variables; IVW, inverse variance weighting; SE, standard error; OR, odds ratio; CI, confidence interval.

p < 0.05 was considered statistically significant.

### 3.2 Causal effect of psoriasis on PBC from MR analysis

**Figure 6:**
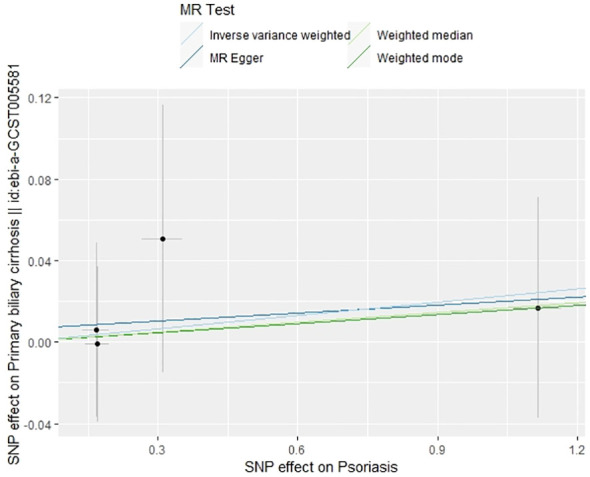
Scatter plot of the effect of psoriasis on PBC.

**Figure 7:**
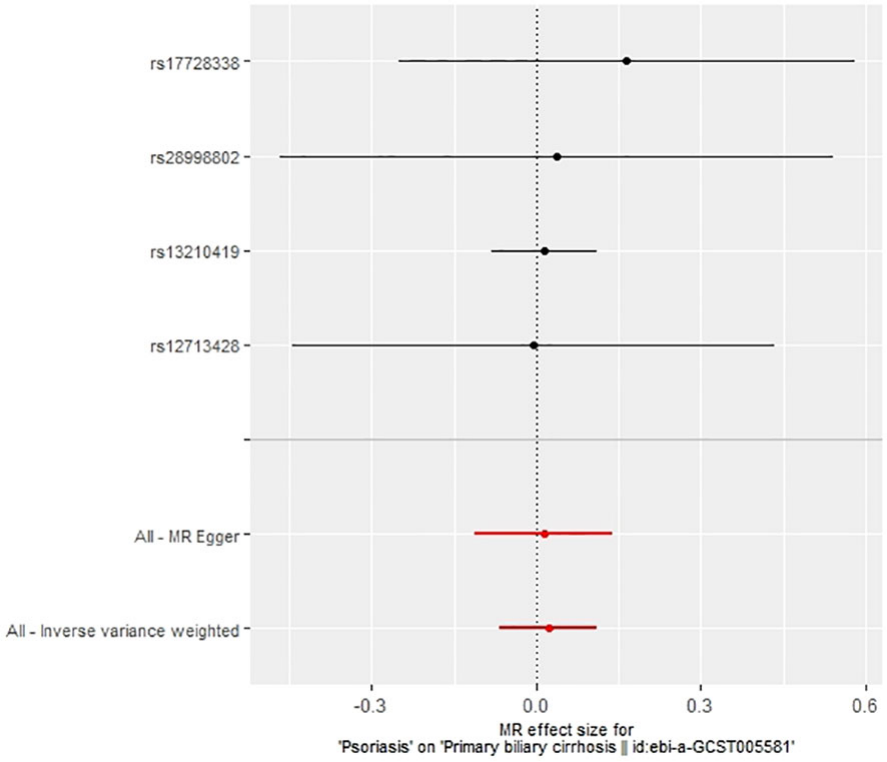
Forest plot of the effect of psoriasis on PBC.

**Figure 8:**
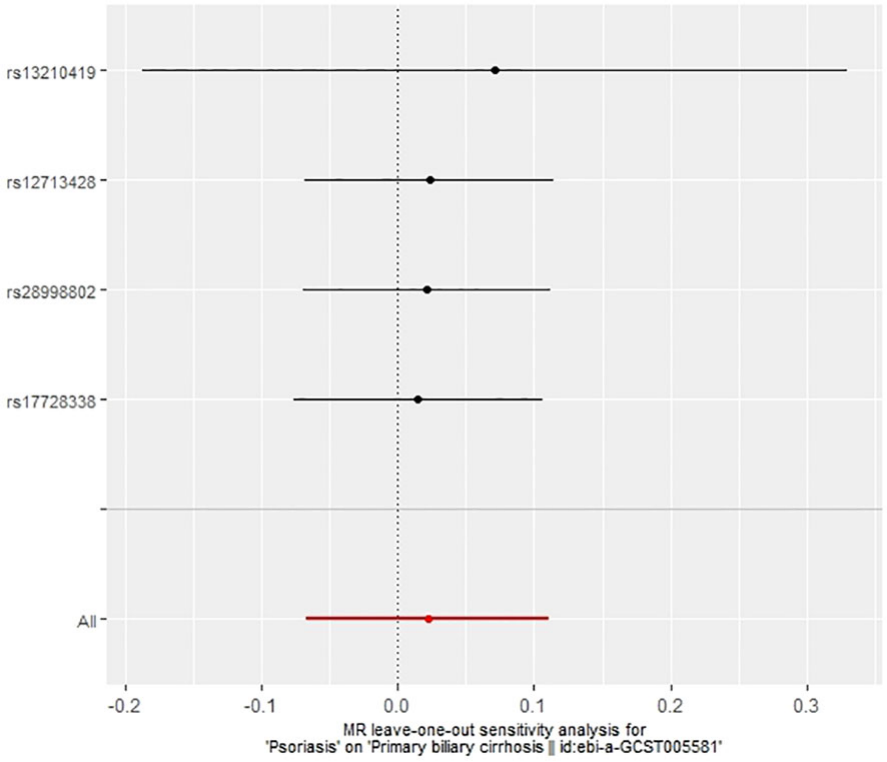
Leave-one-out plot of the effect of psoriasis on PBC.

**Figure 9:**
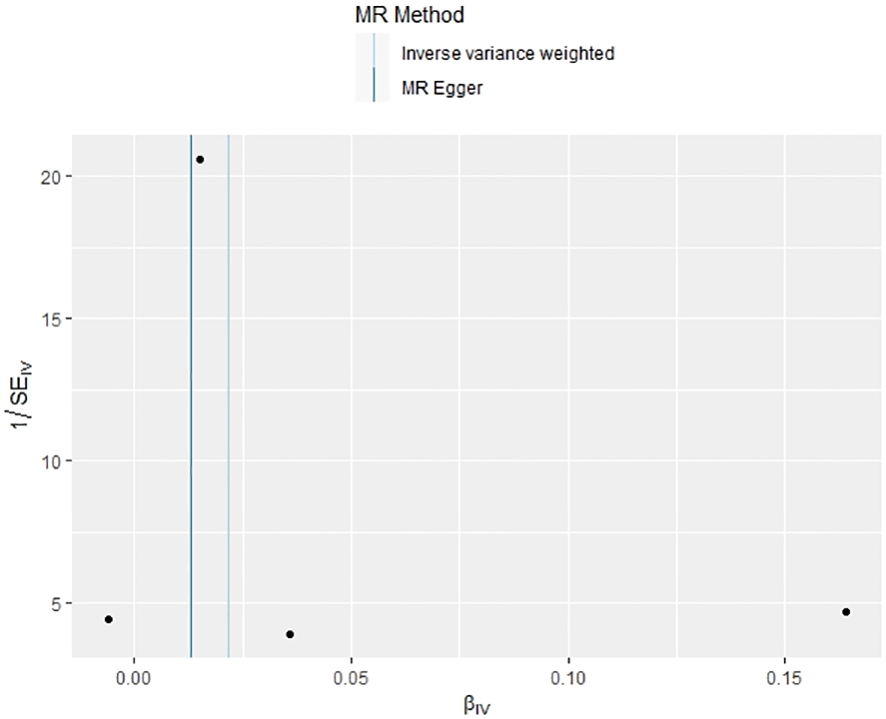
Funnel plot of the effect of psoriasis on PBC.

In our examination of the potential causal effect of psoriasis on the risk of developing PBC, we conducted a two-sample MR analysis with psoriasis as the exposure and PBC as the outcome. The GWAS databases, IV selection methods, analysis approaches, and testing methods were identical to those previously described. Initially, 16 SNPs significantly associated with psoriasis risk (*P*<5×10^-8^, r^2^<0.001) were identified. Of these, 8 SNPs had no corresponding results in the PBC GWAS database. After applying MR-PRESSO, 4 outliers were excluded. The remaining 4 SNPs were then utilized as IVs for MR analysis. Detailed information about these SNPs is provided in **Supplementary Table S2**. The F-statistics for these SNPs all exceeded 10, indicating an absence of weak IV bias. However, none of the methods used—IVW, MR-Egger, weighted median, and weighted mode—demonstrated evidence of a causal relationship between psoriasis and the risk of PBC (*P*>0.05) (**Table 2**). Cochran’s Q test revealed no heterogeneity among the IVs, with a Q-statistic of 0.488 (*P*>0.05). The pleiotropy test using MR-Egger intercept analysis also showed no abnormalities (*P*>0.05) (**Figures 6**–**9**).

**Table 2:** Mendelian randomization estimates for psoriasis on PBC.

Exposure	Outcome	No. of IVs	Methods	Beta	SE	OR (95%CI)	P
Psoriasis	PBC	4	MR Egger	0.013	0.065	1.013 (0.893~1.150)	0.858
			IVW	0.022	0.046	1.022 (0.935~1.118)	0.633
			Weighted mode	0.015	0.049	1.015 (0.923~1.116)	0.779
			Weighted median	0.016	0.047	1.016 (0.927~1.114)	0.734

IVs, instrumental variables; IVW, inverse variance weighting; SE, standard error; OR, odds ratio; CI, confidence interval.

p < 0.05 was considered statistically significant.

## 4 Discussion

PBC is a chronic autoimmune liver disease, which incidence and prevalence are influenced by various factors, including population, region, socioeconomic status, and environmental conditions. Notably, Europe and North America have higher incidence rates of PBC compared to Asia and Africa. Industrialized or polluted areas, along with certain lifestyle choices such as smoking or using nail polish, are associated with a higher risk of developing PBC (20). Patients with PBC may exhibit various skin symptoms like pruritus, pigmentation, butterfly rash, and xanthoma, which can be linked to skin diseases including vitiligo, psoriasis, and rare sterile pustular dermatosis (21). Research indicates that the prevalence of psoriasis in among PBC patients is higher than that in the general population (22). Therefore, it is crucial for doctors treating PBC patients to also monitor their skin manifestations and assess for concurrent psoriasis, as well as environmental factors that might trigger or exacerbate psoriasis, such as streptococcal infections, stress, smoking, obesity, and alcohol consumption (23). For patients suffering from both PBC and psoriasis, interdisciplinary collaboration among medical professionals is essential for timely diagnosis and optimal treatment. A systematic review and meta-analysis published in 2022 identified several medications effective in alleviating PBC-related pruritus (24), including ursodeoxycholic acid (UDCA), methotrexate (MTX), and GSK2330672, an intestinal bile acid transporter inhibitor. These drugs significantly reduced pruritus scores or provided pruritus relief. A study by Hiromasa Ohira et al. reported on six cases of PBC with psoriasis (25), where three patients had plaque psoriasis and three had palmoplantar pustulosis. Notably, all cases of plaque psoriasis were diagnosed after the onset of PBC, and all six patients were treated with UDCA. Additionally, several other studies (26, 27) have observed that UDCA, the first-line treatment for PBC, also positively impacts psoriasis lesions. These findings highlight the potential for similar etiological factors and therapeutic approaches for both PBC and psoriasis, suggesting a close relationship between these two conditions.

PBC and psoriasis, both chronic immune-related diseases, involve abnormal T cell activation and inflammation. Studies highlight the pivotal role of cytokines such as TNF-α, IL-23, and IL-17 in the development and persistence of psoriasis (1). IL-17, crucial in the psoriasis inflammatory cascade, and IL-23, a key regulator of IL-17A production, influence skin conditions by stimulating keratinocyte overproliferation and T cell-mediated inflammation. Targeted inhibition of these cytokines significantly ameliorates skin symptoms and enhances the quality of life in psoriasis patients (28). As components of the “IL-23/IL-17 axis”, these pro-inflammatory cytokines activate inflammatory response effector cells, including neutrophils, macrophages, and fibroblasts. This activation potentially plays a significant role in the pathogenesis of PBC by promoting inflammation (29). However, current literature on the expression differences of IL-23 and IL-17 in PBC patients is limited. Given their roles as key inflammatory factors, IL-23 and IL-17 might impact the skin manifestations observed in PBC patients. Thus, the specific roles of IL-23 and IL-17 in PBC skin manifestations, and the possibility of shared therapeutic targets between PBC and psoriasis, warrant further experimental research. Even if IL-17 and IL-23 have similar roles in both PBC and psoriasis, other genetic or environmental factors might contribute to the differences observed between these diseases. This asymmetry suggests multiple underlying mechanisms that remain to be investigated through comprehensive research.

PBC and psoriasis, while both chronic immune-related diseases, exhibit distinct pathogeneses and target different organs. PBC primarily affects the liver’s small bile ducts, whereas psoriasis predominantly impacts the skin and joints. Additionally, each disease has its unique genetic susceptibilities and risk alleles. Human leukocyte antigen (HLA) alleles are prominent genetic risk factors for autoimmune diseases. PBC’s genetic predisposition is influenced by several HLA alleles, including HLA-DRB1, DR3, DPB1, DQA1, and DQB1. In European populations, DQA104:01 is identified as the most significant risk factor, while DQB103:01 serves as the most robust protective factor (30). Conversely, psoriasis’s genetic susceptibility is a complex multifactorial issue. Significant alleles include HLA-B57, B37, and C06, with C06 being particularly prevalent in psoriasis patients (up to 57.5%). This allele may influence the onset, severity, and subtypes of psoriasis (31). The environmental factors influencing PBC and psoriasis partially overlap, encompassing various infections and allergens. However, notable differences exist. Psoriasis’s development and progression are closely linked to certain drugs, including beta-blockers, lithium salts, synthetic antimalarials, non-steroidal anti-inflammatory drugs, and tetracyclines. These medications can induce or exacerbate psoriasis, or lead to distinct clinical manifestations like psoriatic erythroderma or pustular psoriasis (32). In contrast, PBC’s hallmark marker is the anti-mitochondrial antibody (AMA), a crucial diagnostic criterion. AMA may participate in PBC’s pathologic process by recognizing mitochondrial antigens on bile duct epithelial cells, triggering an autologous immune response from T and B cells, which leads to bile duct inflammation and liver fibrosis (33). Our study indicates that while psoriasis does not increase the risk of PBC, PBC may elevate the risk of developing psoriasis. This finding suggests that the association between these diseases might not be rooted in autoimmune mechanisms. Instead, it points towards the importance of genetic susceptibility and environmental triggers in their correlation.

A case-control study conducted in two British populations examining risk factors for PBC suggested a potential association between psoriasis and PBC (34). However, this link was not statistically significant in a multivariate analysis and might be influenced by other confounding factors. Notably, the study did not specify the onset or diagnosis time of psoriasis, making it difficult to ascertain whether psoriasis preceded PBC. Consequently, establishing a causal relationship between these two diseases remains uncertain. In an individual case reported by Patricija Tomše et al., a 65-year-old patient first developed psoriatic skin lesions in 2016 and was diagnosed with PBC two years later, after starting biological treatments for psoriasis (35). This patient had a history of slightly elevated liver function indicators for years but had not been tested for autoimmune liver disease, suggesting that her PBC might have been undiagnosed for an extended period. PBC often progresses slowly and may present with subtle or overlooked early symptoms, leading to delayed diagnosis. Based on these observations, it is advisable for patients with psoriasis to undergo regular liver function tests and, if abnormalities persist, to be tested for AMA to ensure timely detection and treatment of PBC. Our study concludes that PBC can causally affect psoriasis, but psoriasis does not cause PBC. This finding underscores the importance of considering skin involvement in PBC management. The implications of this study are manifold: 1. It highlights the necessity for healthcare providers to closely monitor the skin conditions of patients being diagnosed and treated for PBC and to promptly identify and manage any concurrent psoriasis. 2. The study advises clinicians to be mindful of the potential impact of PBC treatment drugs on psoriasis, steering clear of medications that might exacerbate or trigger psoriatic symptoms. 3. It emphasizes the need to optimize treatment strategies for patients with both PBC and psoriasis, aiming to reduce adverse drug effects on the liver and improve overall quality of life.

However, our study also has limitations that should be addressed in future research. The data we used lacked detailed demographic information, limiting our ability to perform stratified analyses and understand the disease dynamics across different population segments. Our study focused primarily on European populations, which raises questions about the generalizability of our findings to other ethnic groups. Furthermore, the rarity of PBC has led to limited research on its association with psoriasis, indicating a need for more experimental studies, such as animal models or cellular-level investigations, to validate our findings. Notwithstanding these limitations, our study has significant merits. It is the first to use MR analysis to explore the bidirectional causal relationship between PBC and psoriasis. This method reduces the likelihood of confounding bias and reverse causality affecting the results, a common issue in observational studies. Sensitivity analyses were also performed to ensure the consistency and robustness of our causal estimates and findings.

## 5 Conclusion

Our study reveals that PBC may increase the risk of psoriasis, but psoriasis does not have the same effect on PBC. This identification of a causal association between these disorders provides a new foundation for exploring their common pathogenic mechanisms and potential therapeutic approaches. The insights gained from this study could be valuable in clinical decision-making, potentially improving patient outcomes.

## Data availability statement

The datasets presented in this study can be found in online repositories. The names of the repository/repositories and accession number(s) can be found in the article/**Supplementary Material**.

## Author contributions

WB: Conceptualization, Formal analysis, Methodology, Software, Supervision, Writing – original draft, Writing – review & editing. DZ: Conceptualization, Investigation, Methodology, Software, Writing – original draft, Writing – review & editing. QZ: Data curation, Formal analysis, Methodology, Project administration, Software, Supervision, Validation, Writing – review & editing. FX: Validation, Visualization, Writing – review & editing. FZ: Conceptualization, Formal analysis, Methodology, Software, Supervision, Writing – original draft, Writing – review & editing.
