# Supplementary material for: Primary biliary cirrhosis and psoriasis: a two-sample Mendelian randomization study
Source: Front Immunol. 2024 Jan 4;14:1264554. doi: 10.3389/fimmu.2023.1264554 (PMC10794341; doi:10.3389/fimmu.2023.1264554)
Supplement: Supplementary file 1 [file Table_1.docx]

**Supplementary Materials**

Table S1. Detailed information of instrumental variables utilized in the MR analysis of the causal effects of PBC on psoriasis risk.

| SNP | Effect allele | Other allele | Se | Beta | P | F |
| --- | --- | --- | --- | --- | --- | --- |
| rs79513546 | G | A | 0.212086 | -1.52005 | 7.66E-13 | 51.36789 |
| rs12134279 | T | C | 0.035805 | 0.248421 | 3.97E-12 | 48.13706 |
| rs72678531 | C | T | 0.037073 | 0.479954 | 2.47E-38 | 167.6056 |
| rs10931468 | A | C | 0.042373 | 0.319907 | 4.36E-14 | 56.9987 |
| rs2293370 | A | G | 0.041778 | -0.337293 | 6.83E-16 | 65.18044 |
| rs522127 | C | A | 0.031849 | -0.308974 | 2.98E-22 | 94.11121 |
| rs7665090 | G | A | 0.030708 | 0.229162 | 8.48E-14 | 55.69208 |
| rs6871748 | C | T | 0.035965 | -0.263705 | 2.26E-13 | 53.76215 |
| rs2523882 | T | C | 0.033713 | 0.227136 | 1.61E-11 | 45.39204 |
| rs7775055 | C | T | 0.074902 | 1.11317 | 5.85E-50 | 220.8673 |
| rs35188261 | A | G | 0.043315 | 0.416735 | 6.52E-22 | 92.56255 |
| rs17122453 | A | G | 0.040557 | -0.326839 | 7.71E-16 | 64.94228 |
| rs11065979 | T | C | 0.030841 | 0.183155 | 2.87E-09 | 35.2689 |
| rs1800693 | C | T | 0.03117 | 0.24059 | 1.18E-14 | 59.57663 |
| rs911263 | T | C | 0.035199 | 0.227654 | 9.95E-11 | 41.83115 |
| rs12708716 | G | A | 0.033056 | -0.236736 | 7.97E-13 | 51.29038 |
| rs11117431 | G | A | 0.040941 | -0.23471 | 9.88E-09 | 32.86548 |
| rs8067378 | G | A | 0.030912 | 0.232058 | 6.05E-14 | 56.35438 |
| rs2267407 | A | G | 0.035006 | 0.259283 | 1.29E-13 | 54.86218 |

MR, Mendelian Randomization; PBC, Primary biliary cirrhosis; SNP, single nucleotide polymorphism; SE, standard error.

Table S2. Detailed information of instrumental variables utilized in the MR analysis of the causal effects of psoriasis on PBC risk.

| SNP | Effect allele | Other allele | Se | Beta | P | F |
| --- | --- | --- | --- | --- | --- | --- |
| rs12713428 | C | A | 0.0261 | 0.1694 | 8.11E-11 | 42.12557 |
| rs17728338 | A | G | 0.0439 | 0.3092 | 1.76E-12 | 49.6078 |
| rs13210419 | A | G | 0.0511 | 1.1157 | 1.10E-105 | 476.7087 |
| rs28998802 | A | G | 0.0289 | 0.1672 | 7.41E-09 | 33.47163 |

MR, Mendelian Randomization; PBC, Primary biliary cirrhosis; SNP, single nucleotide polymorphism; SE, standard error.
